# Effects of Auditory Attention Training with the Dichotic Listening Task: Behavioural and Neurophysiological Evidence

**DOI:** 10.1371/journal.pone.0139318

**Published:** 2015-10-06

**Authors:** Jussi Tallus, Anna Soveri, Heikki Hämäläinen, Jyrki Tuomainen, Matti Laine

**Affiliations:** 1 Department of Psychology, Centre for Cognitive Neuroscience, and Turku Brain and Mind Center, University of Turku, Turku, Finland; 2 Department of Psychology, Åbo Akademi University, Turku, Finland; 3 Division of Psychology and Language Sciences, Faculty of Brain Sciences, University College London, London, United Kingdom; University of British Columbia, CANADA

## Abstract

Facilitation of general cognitive capacities such as executive functions through training has stirred considerable research interest during the last decade. Recently we demonstrated that training of auditory attention with forced attention dichotic listening not only facilitated that performance but also generalized to an untrained attentional task. In the present study, 13 participants underwent a 4-week dichotic listening training programme with instructions to report syllables presented to the left ear (FL training group). Another group (n = 13) was trained using the non-forced instruction, asked to report whichever syllable they heard the best (NF training group). The study aimed to replicate our previous behavioural results, and to explore the neurophysiological correlates of training through event-related brain potentials (ERPs). We partially replicated our previous behavioural training effects, as the FL training group tended to show more allocation of auditory spatial attention to the left ear in a standard dichotic listening task. ERP measures showed diminished N1 and enhanced P2 responses to dichotic stimuli after training in both groups, interpreted as improvement in early perceptual processing of the stimuli. Additionally, enhanced anterior N2 amplitudes were found after training, with relatively larger changes in the FL training group in the forced-left condition, suggesting improved top-down control on the trained task. These results show that top-down cognitive training can modulate the left-right allocation of auditory spatial attention, accompanied by a change in an evoked brain potential related to cognitive control.

## Introduction

Training of high-level cognitive capacities such as working memory and top-down attentional control has elicited substantial research interest during the last decade. Of particular interest is whether cognitive training can generalize to untrained tasks that share cognitive mechanisms with the training tasks (for discussion on transfer, see e.g. [1]). Several studies have reported improvements in working memory (e.g. [2–5]) or inhibition of task-irrelevant information [6] through computerized training, along with transfer to untrained tasks. However, many cognitive training studies have also been criticized for methodological shortcomings, such as for not employing a control group, or only measuring the control group’s performance twice at different time points with no active placebo intervention in between [7]. The present study, using an active control group, focused on the neurocognitive effects of training of auditory attention via dichotic listening.

In the non-forced (NF) variant of the dichotic listening (DL) task (e.g. [8]), the participant is presented with two meaningless syllables simultaneously to both ears and instructed to report whichever syllable he or she heard best. In the forced-attention variant of the DL task, the participant is instructed to report either the left-ear syllable (forced left (FL) condition) or the right-ear syllable (forced right (FR) condition). The forced-attention conditions of the DL task, especially the condition where auditory attention is directed to the left ear, is considered to tax executive resources. Normally, in the NF condition right-handed participants demonstrate a tendency to report more right-ear syllables in a verbal DL task, which is called the right-ear advantage (REA). This advantage is thought to be a result of the structural properties of the auditory and language systems in the brain [9]. During dichotic listening, auditory input from either ear crosses over to the contralateral cerebral hemisphere, with ipsilateral inputs being automatically inhibited. Thus right-ear auditory input has a privileged access to left hemisphere language processing areas, resulting in a REA for recognition of verbal stimuli. In the FL condition, participants are instructed to attend only to the left-ear stimuli, thus forcing them to counteract the REA. To do this, participants have to actively orient their attention to the left-ear syllables while inhibiting the right-ear stimuli, which are usually available with little or no effort. There is evidence for both facilitation of attended ear responses and inhibition of intrusions from the non-attended ear. The latter is mainly responsible for changes in the proportion of right and left ear responses in the FL and FR conditions relative to the NF condition [10]. Especially the FL condition requires the participant to engage in top-down attentional control and inhibition of prepotent responses, as it involves a conflict between the bottom-up stimulus-driven processes favouring a REA, and the instruction to report left ear stimuli [11–13].

In a recent study [14] we demonstrated changes in performance on the forced attention DL task as a result of a 4-week DL training regime. In that study, participants were randomly divided into four groups. Of particular interest for the present purposes were the two groups which trained DL either with the FL instruction or with the NF instruction. Participants who trained with the FL instruction demonstrated more left-sided responses in the standard NF DL task after training. They also showed evidence for treatment generalization to the so-called auditory go/no-go spatial attention task [15]. This task requires the participant to simultaneously monitor two verbal spoken streams of digits, one presented to the left and one to the right ear. This task bears resemblance to the forced attention DL task, but does not involve any instruction to focus on input to either ear over the other. Rather, the participants are instructed to report all target digits they hear regardless of ear. Before training, participants tended to report only the right-sided target on such trials where targets were presented simultaneously to both ears (i.e. bilateral trials), reminiscent of DL REA. After 4 weeks of training, the participants who had trained FL reported more often the left-sided targets on these trials, suggesting a transfer effect. Taken together, these findings suggest an increased tendency to attend to the left auditory space after 4 weeks of FL DL training.

Training executive control has, depending on the experimental setup, been manifested as increases and decreases in the activity of certain brain regions. Most commonly reported are effects on parietal and frontal regions (e.g. [2, 5, 16–20]), which are part of the attentional neural network [21]. Generally, cognitive capacity and brain activity in task-relevant areas seem to be positively correlated (for a review on working memory tasks, see [22]). However, several studies have also shown decreased activation after training, being attributed to increased cortical efficiency [6]. Many parallel behavioural and neural changes may occur during training of higher-level cognitive domains such as executive control [22]. Therefore, one should probably not expect any simple neural correlates of improved executive functions following different training regimes.

Electrophysiological studies suggest that executive training may lead to changes in N1, N2, P2 and P3 amplitudes evoked by visuospatial stimuli [23–26]. The N1 waveform is generated by stimulus onset or offset, and is modulated by several physical properties of the stimulus [27]. The N1 is also modulated by attention, as enhanced N1 amplitudes are seen for attended stimuli, and the latter (~150 ms) part of N1 is also closely related to the mismatch negativity [27–28]. The N1 and P2 responses covary, but can be dissociated in the auditory domain by experimental manipulations [29] or cerebral lesions [30], and therefore should not be considered to reflect the same cerebral processes. Like N1, the P2 is evoked also by unattended stimuli, but is modulated by attention, as attended auditory stimuli evoke smaller P2 responses [29]. The functional significance of the auditory P2 is unclear, but it has been suggested to reflect stimulus categorization processes and possibly withdrawal of attention from irrelevant/non-target stimuli. Later N2–P3 ERPs are thought to relate more to top-down processes, including inhibitory control, processing stimulus conflict and error monitoring [24–25, 31].

In the visual domain, posterior N1 changes have been found after WM training, including decreased N1 amplitudes that correlated with improved WM accuracy [23] and increased amplitudes for non-targets in a transfer task, suggested to be related to improved sustained attention [26]. Increased frontal P2 to non-targets was found after visual WM training [26] and increased P2 in a visual go/no-go task after 4 weeks of musical training [25]. In clinical training interventions focused on top-down attentional control (relative to anxiety inducing or painful stimuli), training effects were manifested as changes in later ERPs (increased N2 amplitude, decreased P2 and P3 [24], and as correlations of P2, P3, N400 and P600 amplitudes with behavioural improvements [32]. Visuospatial WM training has also increased dynamic connectivity of the superior parietal lobule as evaluated by TMS/EEG and decreased contralateral delay and search activities, possibly because of improved allocation of attentional resources [33].

Functional neuroimaging studies on DL have shown that the cognitively more demanding FL condition activates especially the left middle frontal gyrus [34], the left inferior prefrontal gyrus, and the caudate nucleus [35]. Stronger callosal interhemispheric connections are associated with reduced auditory laterality in the NF condition, most likely because of better processing of left-ear input in the left hemisphere. Callosal connectivity is also related to top-down control in DL, although fewer studies are available on this topic [36]. Hemispheric asymmetries are reflected also in electrophysiological studies on DL. The left temporal N1 to dichotic consonant-vowel syllables has approximately 15 ms shorter latency than the right temporal N1 [37]. Verbal stimuli presented to the right ear evoke a shorter latency ERP waveform on both hemispheres compared to stimuli presented to the left ear, the difference being most pronounced over the left hemisphere [38]. In addition, the late negative potential (440–519 ms) has shorter latency on trials resulting in REA than on trials resulting in left-ear advantage (LEA) [39].

The present study had two main aims. Firstly, it aimed to replicate the behavioural results of the earlier study [14]. Secondly, we recorded the participants’ ERPs during pre- and post-training DL performances to explore the neurophysiological correlates of the training effects. Our hypotheses were that a) the training improves overall DL performance (more correct responses altogether and less intrusions from the non-attended ear), with the FL training especially facilitating FL performance, b) the training generalizes to the untrained auditory go/no-go spatial attention task as shown by increased response rate to left-sided stimuli relative to right sided stimuli in the bilateral stimulation condition, and c) the behavioural training effects are accompanied by ERP changes in the N1–P2 complex and in the N2 potential, as these potentials are related to both the REA and attention processes. These hypothesized ERP changes would reflect enhanced perceptual processing of stimuli (cf. [25–26, 37], and top-down control [24–25], respectively.

## Materials and Methods

### Ethics statement

All participants gave their written informed consent prior to participation. The study was conducted in compliance with the declaration of Helsinki. The study was approved by the Centre for Cognitive Neuroscience institutional review board.

### Participants

Participants (n = 26, 14 males) were 20–34 year-old, neurologically healthy volunteers. All participants were native Finnish speakers with normal hearing acuity (hearing threshold 20 dB or better in both ears at 250, 500, 1000 and 2000 Hz). All participants were right-handed according to the Edinburgh Handedness Inventory score [40]. In the initial assessment, all participants had the expected REA in the NF DL. The participants were randomly divided into two groups (FL and NF training groups) with the restriction that the sex ratio was the same in both groups. No differences in mean age, digit span scores or handedness scores were found between the groups (t-tests p > .05). A small financial compensation was paid for participation.

### Experimental tasks

A computerized Finnish version of the consonant-vowel DL task [9] was used. Task stimuli were the syllables [ba], [da], [ga], [pa], [ta] and [ka] read by a male voice with even intonation and intensity, presented simultaneously to both ears through earphones at 70 dB. The participant was either instructed to report whichever syllable he/she heard the best (NF condition), or the syllables presented through the left earphone (FL condition), or the syllables presented to the right earphone (FR condition). Each condition included 36 trials, i.e. all possible combinations were used once.

As transfer tasks, we used the Simon task [41], the landmark task [42], and the auditory go/no-go spatial attention task [14–15]. These tasks were specifically selected to test the participants’ capacity for stimulus inhibition and spatial attention with both auditory and visual stimulus material. All transfer tasks and the DL task were run on a computer using Presentation (version 14.0; NeuroBehavioral Systems, CA, USA).

In the *Simon task*, the participant was presented with a red or blue box in either the left or the right side of the computer screen, and was instructed to respond as quickly as possible by pressing the left-sided response key on the keyboard when a blue box was presented, and the right-sided response key when a red box was presented. The box was visible until the participant gave a response. Then a blank screen with only a fixation mark was displayed for 5 s before next trial. The trials were either congruent (e.g., target presented to the left and left-sided response required) or incongruent (e.g., target to the left but right-sided response required). One hundred trials were presented. Participant’s reaction times and number of correct and incorrect responses were recorded separately for the congruent and incongruent trials.

In the *landmark task*, the participant was presented with 120 trials of horizontal white lines bisected close to the centre with a white vertical line, against a black background. The participant’s task was to judge whether the left or right side of the bisected line was longer. The horizontal line could be bisected either 0.7, 1.4, 2.1, 3.6, or 6.4% of the whole horizontal line length off to the left or right of the true centre, or exactly at the true centre. The order of the trials was randomized. The bisected line was displayed for a maximum of 2 s (trial ended when a response was given). In-between trials, a white rectangular box was displayed at the centre of the computer screen for 1 s, covering the entire area of the trial stimulus. Responses were given by pressing the left or right response button on the keyboard. The task was arranged in two blocks, with half of the responses being given with the left and the other half with the right hand. The total number of left (i.e. “left side of the bisected line is longer”) and right responses were summed up separately, irrespective of their correctness.

In the *auditory go/no-go spatial attention task*, the participant heard through earphones two separate streams of spoken digits (1–9), one to the left and the other to the right ear. The participants were instructed to press the left response key as quickly as possible every time a specified left ear target digit was presented to the left ear, and the right response key every time a specified right ear target digit was presented to the right ear. The targets could be presented to the left or right ear either one at a time or simultaneously. The participants completed two blocks of this task, the easier with inter-stimulus intervals of 200–1000 ms, and the harder one with intervals of 150–650 ms. Altogether 480 unilateral or bilateral sound stimuli were presented, including 80 unilateral and 40 bilateral target stimuli. Reaction times and the number of correct responses were recorded separately for the left and right sided targets, and also separately for single unilateral targets and bilateral simultaneously presented targets.

Additionally, during the initial testing session the participants' working memory performance was measured using the Wechsler Adult Intelligence Scale III (WAIS-III) digit span subtest. This was used to control for the baseline WM capacity of the two groups.

### Training procedure

All participants completed the pre-training behavioural measurement, pre-training EEG measurement, a four-week training period (randomized into one of the two training groups), and post-training EEG and behavioural measurements. The pre-training behavioural measurement included the DL task, the transfer tasks, and the digit span test. The post-training measurement included the same battery except the digit span test. During the pre- and post-training EEG measurements, the participants performed the forced attention DL task with the NF, FL and FR instructions, with 108 trials in each condition. Behavioural DL methods were replicated from [14]. Behavioural data were not collected during the EEG measurements.

The auditory attention training included four 25-minute training sessions per week for four weeks. During the training sessions, the participants performed the DL task with either the FL (the FL training group) or the NF (the NF training group) instruction. During training, the DL task was presented in blocks of 36 trials, and the participants were allowed short breaks between the blocks. A single session included approximately 300 trials. The training was conducted in a laboratory setting.

### EEG measurement

EEG was recorded using electrodes attached to a cap (Easycap GmbH, Herrsching-Breitbrunn, Germany) according to the international 10/20 system. The electrode sites were Fp1, Fp2, F3, F4, F7, F8, C3, C4, P3, P4, T3, T4, T5 and T6. The reference electrode was placed on the tip of the nose and FCz was used as ground. Vertical eye movements were monitored using an electrode placed under the right eye and horizontal eye movements were monitored using an electrode placed on the outer canthus of the right eye. The impedance of the electrodes was kept below 5 kΩ. The EEG was amplified with SynAmps^2^ (Compumedics Neuroscan, NC, USA) using a 500 Hz sampling rate. The EEG was filtered off-line using a 1–25 Hz band-pass filter. The data were split into epochs -150–500 ms relative to stimulus onset and the -150–0 ms interval was used for baseline correction. Trials with artefacts larger than 70 μV were rejected, and the remaining trials were averaged. Data from different electrodes were pooled to form four regions of interest (left anterior, LA: F3, F7, C3; left posterior, LP: P3, T3, T5; right anterior, RA: F4, F8, C4; right posterior, RP: P4, T4, T6). Mean voltages were calculated for each region of interest (ROI) from time windows 90–140 ms (N1), 165–215 ms (P2) and 260–310 ms (N2). The EEG data were analyzed with BrainVision Analyzer 2 (Brain Products, Gilching, Germany).

### Statistical analyses

T-tests were used to evaluate possible intergroup pre-training differences on the mean age, handedness score, and the WAIS III forward and reverse digit span, and no significant differences were found. Neither did we observe any significant pre-training differences in DL performance between the groups.

The DL data were analysed by separate mixed-model 2 × 2 × 2 (time × ear × group) ANOVAs for correctly reported syllables in each attention condition. The stimuli included congruent syllable pairs (e.g. [ba]-[ba], 6 such trials in each behavioural DL block and 18 in each EEG DL block) to control that the participants could classify the syllables correctly, but these trials were not included in the statistical analyses.

The EEG data were analysed separately in each attention condition (NF, FL, FR) using 2 × 2 × 2 ANOVAs (time × ROI × group). Separate ANOVAs were performed with N1, P2, and N2 mean amplitudes.

Greenhouse-Geisser correction was used when the sphericity assumption was violated (uncorrected degrees of freedom with ε-values are reported). T-tests with Bonferroni correction were used for post-hoc comparisons. Results with significant effects of time (before—after training) are reported for the experimental tasks.

## Results

### Dichotic listening task behavioural data

**Fig 1:**
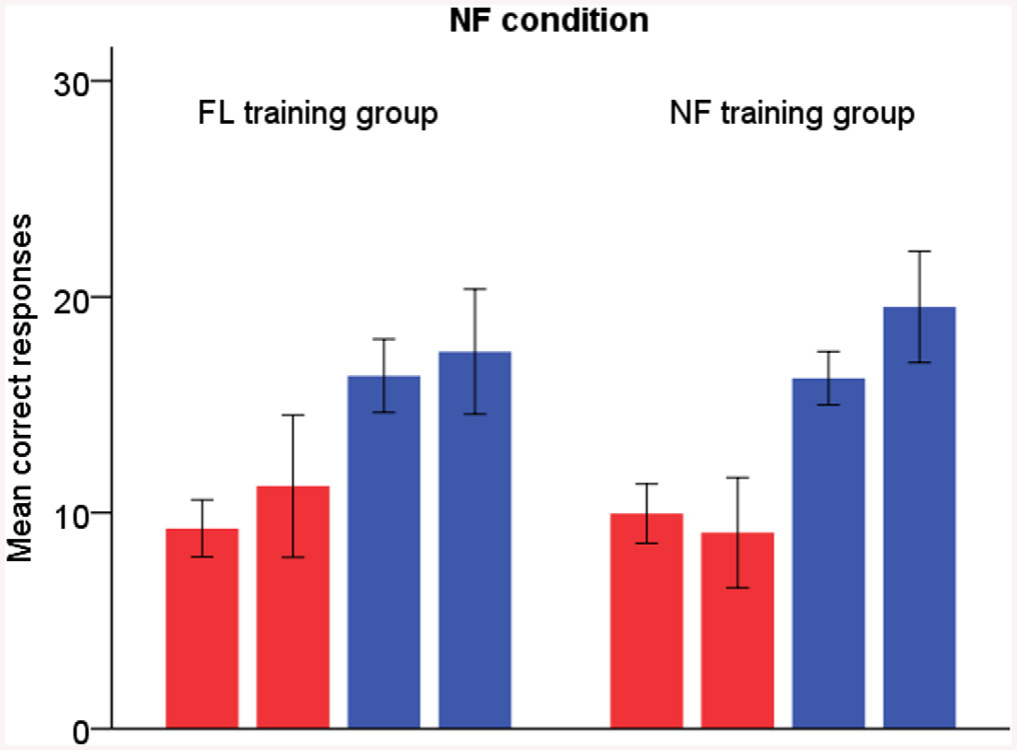
NF condition. Red bars = left-ear responses, blue bars = right-ear responses. The left-sided bar of each two bars of the same colour represents pre-training results and the right-sided bar post-training results. Error bars display 95% confidence interval for mean.

In the NF condition, both groups reported more syllables correctly after training (time F_1,24_ = 55.95, p < .001, η^2^_p_ = .70). The FL training group tended to increase their left-ear responses, while the NF training group showed an opposite trend (see Fig 1, change in the left-ear responses 17 ± 45% in the FL training group vs. -10 ± 38% in the NF training group, and change in the right-ear responses 6 ± 20% in the FL training group vs. 20 ± 21% in the NF training group). This was similar to what was found in our previous study, approaching significance in the present data (time × ear × group F_1,24_ = 3.70, p = .066, η^2^_p_ = .13).

**Fig 2:**
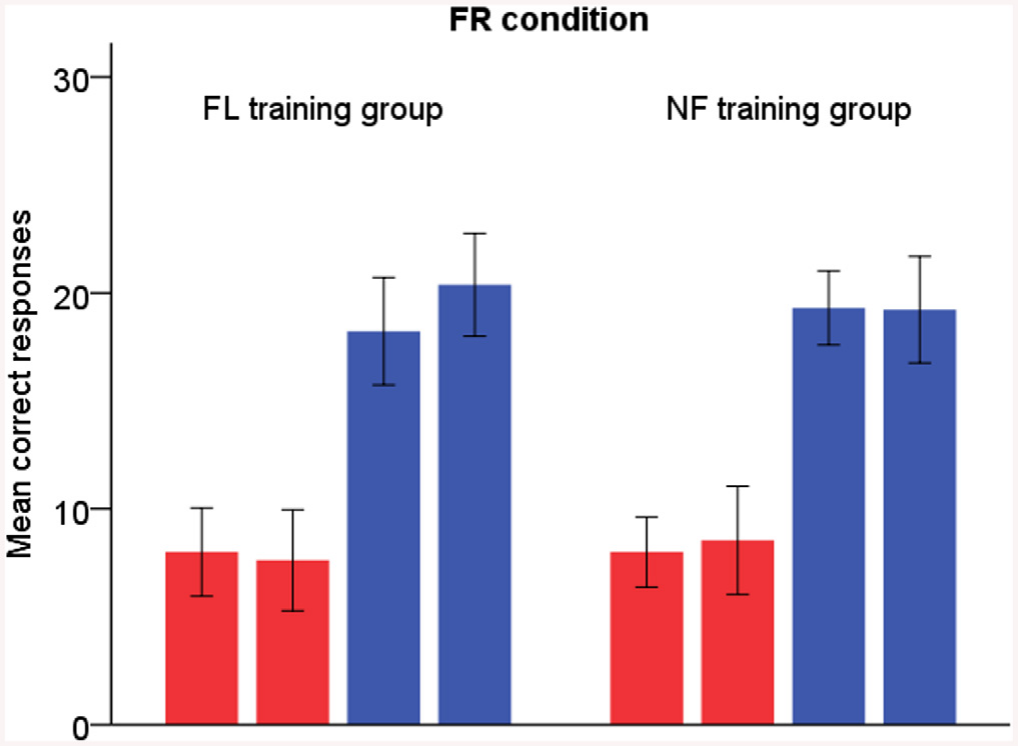
FR condition. Red bars = left-ear responses, blue bars = right-ear responses. The left-sided bar of each two bars of the same colour represents pre-training results and the right-sided bar post-training results. Error bars display 95% confidence interval for mean.

In the FR condition (Fig 2), both groups reported more syllables correctly after training (time F_1,24_ = 6.84, p = .015, η^2^_p_ = .22), but no other training effects were found.

**Fig 3:**
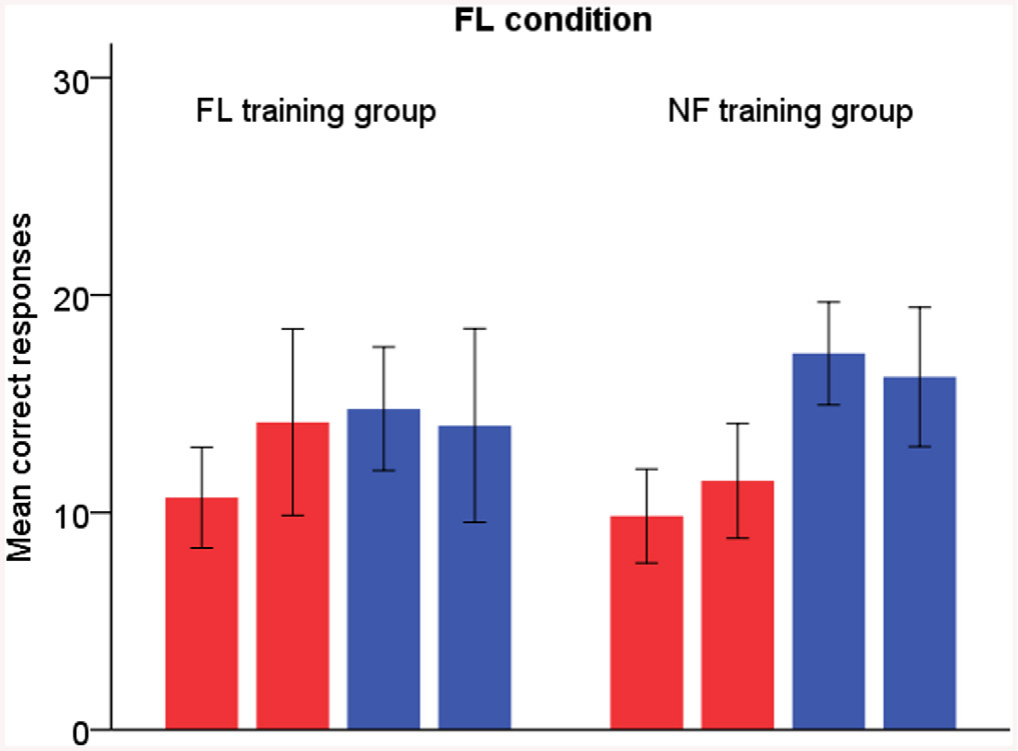
FL condition. Red bars = left-ear responses, blue bars = right-ear responses. The left-sided bar of each two bars of the same colour represents pre-training results and the right-sided bar post-training results. Error bars display 95% confidence interval for mean.

In the FL condition, both groups also reported more syllables correctly after training (time F_1,24_ = 7.40, p = .012, η^2^_p_ = .24). We expected the FL training group to increase their correct responses more than the NF training group as in the previous study. The mean values pointed to this direction, but the effect was not quite statistically significant (time × group F_1,24_ = 3.29, p = .082, η^2^_p_ = .12; see Fig 3). Both groups also showed a tendency to a greater increase in left-ear responses than right-ear responses (time × ear F_1,24_ = 3.69, p = .067, η^2^_p_ = .13).

### Transfer tasks

No significant time × group interactions were found in the transfer tasks. To test our hypothesis in the auditory go/no-go spatial attention task, separate analyses were done for unilaterally and bilaterally presented targets, with the number of correct responses as the dependent variable. Mixed model 2 × 2 × 2 × 2 (time × task difficulty × response side × group) ANOVAs were calculated.

The interaction of time × response side × group which we found for bilateral targets in our previous study was not replicated (F_1,24_ = 0.35, p = .558, η^2^_p_ = .01). For unilateral targets, both groups increased their left-sided responses more than right-sided responses from pre- to post-test (time × response side F_1,24_ = 7.62, p = .011, η^2^_p_ = .24).

### EEG—NF condition

**Fig 4:**
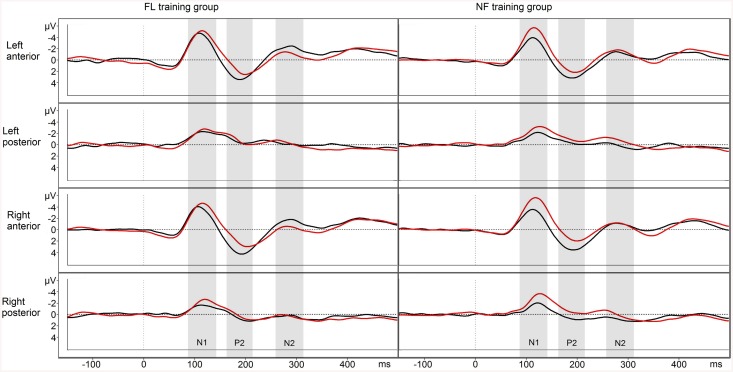
NF condition EEG responses. Pre-training waveform in red and post-training waveform in black. Negative plotted up.

The N1 amplitude was decreased after training in both groups (time F_1,24_ = 14.219, p = .001, η^2^_p_ = .37) (see Fig 4). The P2 amplitude was increased after training in both groups (time F_1,24_ = 25.385, p < .001, η^2^_p_ = .51), and this increase was more pronounced on the anterior ROIs (time × ROI F_3,72,.514_ = 5.750, p = .011, η^2^_p_ = .19). The anterior N2 amplitude was also increased after training in both groups (time × ROI F_3,72,.682_ = 12.027, p < .011, η^2^_p_ = .33).

The amplitude values of N1, P2 and N2 for both groups before and after training are displayed in Table 1.

**Table 1:** Mean amplitudes (SD) before and after training. Mean (SD) separately for each DL condition and both groups. Amplitudes are averaged across all ROIs.

		N1	P2	N2
NF	FL training group before	-3.13 (1.72)	0.88 (2.59)	-0.38 (1.34)
	after	-2.63 (0.97)	1.70 (2.65)	-0.87 (1.42)
	NF training group before	-3.67 (1.38)	0.42 (1.15)	-0.76 (1.72)
	after	-2.26 (1.29)	1.58 (0.94)	-0.25 (1.22)
FR	FL training group before	-2.89 (1.71)	0.37 (2.44)	-1.45 (1.13)
	after	-2.45 (0.85)	1.68 (1.78)	-1.14 (1.49)
	NF training group before	-2.80 (1.23)	0.41 (1.52)	-1.02 (1.57)
	after	-2.39 (1.18)	0.88 (1.30)	-0.92 (1.09)
FL	FL training group before	-2.70 (1.11)	0.86 (2.45)	-0.83 (1.04)
	after	-2.62 (1.07)	1.29 (2.04)	-1.43 (0.89)
	NF training group before	-2.99 (1.36)	-0.05 (1.41)	-0.88 (1.42)
	after	-1.93 (1.31)	0.62 (1.38)	-0.83 (1.15)

### EEG—FR condition

**Fig 5:**
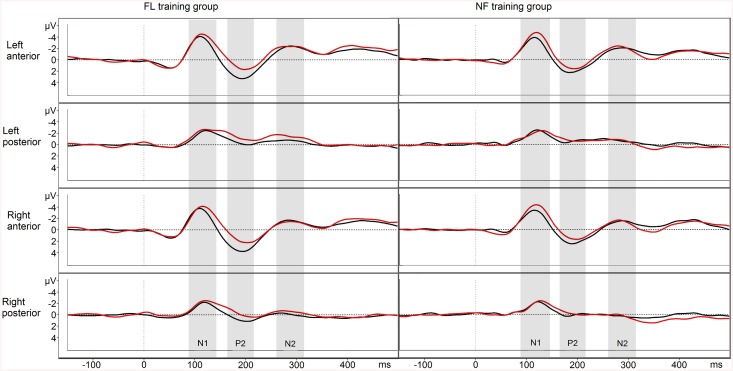
FR condition EEG responses. Pre-training waveform in red and post-training waveform in black. Negative plotted up.

The N1 was decreased on the anterior ROIs after training (time × ROI F_3,72,.659_ = 3.913, p = .027, η^2^_p_ = .14). The P2 amplitude was increased after training (time F_1,24_ = 10.228, p = .004, η^2^_p_ = .30). This increase was more pronounced on the anterior ROIs (time × ROI F_3,72,.724_ = 4.587, p = .013, η^2^_p_ = .16) (see Fig 5).

### EEG—FL condition

**Fig 6:**
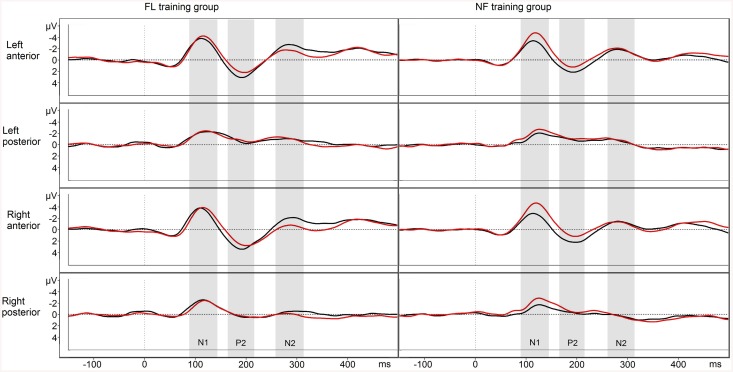
FL condition EEG responses. Pre-training waveform in red and post-training waveform in black. Negative plotted up.

The N1 was decreased after training (time F_1,24_ = 5.570, p = .024, η^2^_p_ = .19), especially on the anterior ROIs (time × ROI F_3,72,.729_ = 4.154, p = .018, η^2^_p_ = .15), and there was a trend of N1 amplitudes decreasing more in the NF training group than in the FL training group (time × group F_1,24_ = 4.128, p = .053, η^2^_p_ = .15). The P2 amplitude was increased after training on the anterior ROIs (time × ROI F_3,72,.595_ = 10.294, p < .001, η^2^_p_ = .30). The N2 amplitude was increased after training on the anterior ROIs (time × ROI F_3,72,.616_ = 4.491, p = .019, η^2^_p_ = .16). This increase was observed especially in the FL training group (time × ROI × group F_3,72,.616_ = 3.676, p = .037, η^2^_p_ = .13). Follow-up ANOVAs separately for each group showed no significant effects for the NF training group (ps >.5), but a time × ROI interaction for the FL training group (F_3,36,.649_ = 6.384, p = .006, η^2^_p_ = .35). This interaction was followed with post-hoc t-tests, showing that the FL training group’s right anterior N2 was increased from pre- to post-test (t_12_ = 4.272, p = .004), while other differences were non-significant after Bonferroni correction (see Fig 6).

## Discussion

The first aim of the present study was to try to replicate the findings of Soveri et al. [14] who observed a relative leftward shift in the allocation of auditory spatial attention following DL training with the FL condition, i.e. more left-ear responses in relation to right-ear responses in the NF condition than before training. They also reported an overall trend for more correct syllables post training in the FL condition. In the present study, similar behavioural patterns were found in the NF and FL conditions, and the effect sizes found in both studies were also very similar, although in the present experiment the effects were only nearly significant, possibly due to a smaller sample size. With regard to transfer effects, Soveri et al. [14] observed that in the auditory go/no-go transfer task their FL training group displayed a trend for more often responding to the left-sided targets in bilateral target presentation after training than before training. At the same time, right-sided responses decreased among participants receiving FL training and increased in those who did not receive FL training. However, the present study found no transfer to the auditory go/no-go spatial attention task in the FL training group. Instead, for unilateral targets (i.e. target digit presented only to either the left or the right ear), both groups increased their correct left-sided responses.

In the EEG data, changes as a function of training were found in all examined ERPs. For both groups, N1 amplitudes were decreased in all conditions after training, with greater changes over the anterior regions (in the FL and FR condition), and a trend for a more pronounced amplitude decrease in the FL condition for the NF training group than for the FL training group. The P2 amplitudes were increased in all conditions after training, especially on the anterior regions. The N2 was increased on the anterior regions in the NF and FL conditions after training. In the FL condition, the FL training group showed a greater increase in N2, mostly on the right anterior ROI. However, inferences about the neural sources of the ERP effects must be made with caution as the responses from different ROIs reflect summation of potentials from a wider area. Nevertheless, anteriorly biased training-induced ERP changes would concur with the view that the training affected attentional control mechanisms [43–44].

As increased N1 amplitudes are related to attending to stimuli [27–28], the decrease in the N1 amplitudes after training might indicate less attentional resources devoted to the stimuli, possibly due to improved early processing of the stimuli, increased familiarity with the task, or perceiving the task as easier and less demanding than before training. In visual WM training studies, decreased N1 amplitudes have been shown to correlate with improved accuracy [23]. Increased P2 across all conditions may also be interpreted to reflect improvements in early perceptual processing of the stimuli (cf. [23, 26] for studies in the visual domain and [45]). However, it has been argued that sufficient exposure to specific auditory stimuli by itself leads to enhanced P2 and the amount of change in P2 is unrelated to training gains [46–47]. The N2, on the other hand, is associated with top-down attentional control [24–25, 31], and both groups increased N2 amplitude in NF and FL, but the FL training group showed a larger N2 amplitude increase in the FL condition. This finding is in line with our expectation that FL training would benefit top-down control more than NF training. Even though the present behavioural results are inconclusive in this respect, the previous results [14] pointed in this direction.

In this study we were able to partially replicate our previous findings on the effects of auditory attention training with the FL task. As the present training procedure was identical with our previous one, reasons for the different outcomes must be sought elsewhere. One notable methodological difference is the inclusion of the EEG session in the present study. During EEG, all participants performed DL in all attention conditions, giving the present NF training group somewhat more practice in forced attention DL prior to the actual training period. Post-training EEG measurement could however not affect the behavioural results, as it was done after the post-training behavioural tests. The age range and educational background of the participants in the present and our earlier study were similar, but the sex ratio differed. The present study evidenced a slight male predominance (14 out of 26 participants) while in the previous study more females participated (38 out of 50 participants). Group-wise male-female distributions were even in both studies, so this should not explain differences between groups, unless men and women reacted to the auditory attention training differently. We are unaware of any evidence supporting this possibility. On average, men have a slightly stronger REA in verbal DL, but this effect is quite small (effect size 0.054 based on a meta-analysis [48]) and thus unlikely to cause such a difference in the results of our present and previous studies.

We employed three attentional/executive transfer measures, including a task that indicated FL training-related behavioural effects in our previous study. However, the present study failed to show any transfer effects specific to the type of training. The lack of transfer effects may be interpreted so that training did not affect the participants’ capacity for attention and inhibitory control. Consequently, improvements in the criterion task (DL) should rather be explained with more task-specific effects, such as stimulus familiarity and more automatized responses. Another reason for the lack of evidence for transfer in this study could be the smaller sample size compared to the previous study.

Together with our previous training study [14], the results indicate that allocation of auditory spatial attention in the left vs. right auditory hemispace can be modulated by top-down cognitive training. This was accompanied by changes in evoked brain potentials, including the N2, which has previously been associated with cognitive control. However, it appears that the training effects are rather task-specific and not readily generalizable to other auditory tasks.

## Supporting Information

S1 Dataset SPSS data sheet containing the behavioural and EEG data. Please refer to the SPSS variable list for variable descriptions.
(SAV)
